# Association of Dyslipidemia With Dermatological Manifestations in Patients With Type 2 Diabetes Mellitus

**DOI:** 10.7759/cureus.86030

**Published:** 2025-06-14

**Authors:** Syeda Shahmoona Tirmizi, Syed Mamoon Akhtar, Aisha Naz Rajput, Adnan Anwar, Umair Tariq, Atif A Hashmi

**Affiliations:** 1 Dermatology, Hamdard Medical University, Karachi, PAK; 2 Internal Medicine, Shaikh Tahnoon Bin Mohammed Medical City, Abu Dhabi, ARE; 3 Internal Medicine, Amana Healthcare, Abu Dhabi, ARE; 4 Physiology, Hamdard College of Medicine and Dentistry, Karachi, PAK; 5 Internal Medicine, Sindh Government Hospital, Karachi, PAK; 6 Internal Medicine, Army Medical College, Rawalpindi, PAK; 7 Pathology, Liaquat National Hospital and Medical College, Karachi, PAK

**Keywords:** dermatological manifestations, dyslipidemia, symptoms, type 2 diabetes mellitus, xerosis

## Abstract

Objectives

Type 2 diabetes mellitus (T2DM) is frequently related with various cutaneous manifestations, which may be influenced by comorbid conditions such as dyslipidemia. Hence, this study compared the demographic to match clinical and dermatological profiles of T2DM patients with and without dyslipidemia.

Methodology

This cross-sectional study was conducted at a secondary care hospital using a non-probability convenient sampling technique over six months. A total of 280 T2DM patients aged 40-65 years were included in this study. Patients were distributed into two equal groups: Group A (with dyslipidemia) and Group B (without dyslipidemia). Data on demographics, clinical history, lifestyle factors, treatment regimens, and dermatological findings were collected using structured questionnaires, physical examinations, and laboratory tests. A Chi-square test and a Mann-Whitney test were applied to examine the association of dermatological symptoms in T2DM. A p-value < 0.05 was reflected as statistically significant.

Results

The study findings showed that a significant gender disparity was observed: all patients in Group A were male subjects at 140 (100%), while Group B were predominantly female subjects at 88.6% (p<0.001). A larger proportion of Group A had diabetes for more than five years, 47 (33.6%) compared to Group B at 14 (10.0%), (p<0.001), and more frequent use of insulin and oral hypoglycemic agents (p=0.001). Xerosis with fissured skin was significantly more common in Group B at 109(77.9%), (p<0.001). The presence of bullae was also significantly greater in Group B at 54(38.6%) as compared to Group A at 25 (17.9%) (p<0.001). Although acanthosis nigricans showed no significant difference, it was slightly more prevalent in Group B.

Conclusion

This study concluded that the dermatological manifestations were common among T2DM patients, with notable differences based on dyslipidemia status. Patients without dyslipidemia showed a higher prevalence of xerosis, ichthyosis, callosities, and bullae, whereas paresthesias were more frequently observed in those with dyslipidemia.

## Introduction

Diabetes mellitus is associated with a wide variety of skin manifestations, varying from harmless cosmetic issues to serious complications. These dermatologic changes can provide important insights into a patient’s current or previous metabolic condition. Detecting such skin signs may aid in diagnosing diabetes or indicate long-term uncontrolled glycemic index. Most skin complications linked to diabetes result from prolonged high blood sugar levels, the buildup of advanced glycation end products (AGEs), vascular abnormalities, immune system dysfunction, and nerve damage [1].

Skin disorders are observed in approximately 79.2% of individuals with diabetes [2]. In a research study involving 750 diabetic patients, the most frequently reported skin conditions included cutaneous infections (47.5%), xerosis (26.4%), and inflammatory dermatoses (20.7%) [2]. Furthermore, cutaneous manifestations tend to occur more commonly in patients with type 2 diabetes mellitus (T2DM) in comparison with type 1 diabetes.

Dyslipidemia is a significant contributor to cardiovascular disorders in individuals with T2DM. It is typically marked by raised plasma triglycerides (TG), decreased high-density lipoprotein (HDL) cholesterol, and increased low-density lipoprotein (LDL) cholesterol alterations commonly observed in diabetic patients [3]. While triglycerides comprise various molecular species and lipoproteins are associated with numerous lipid classes, the specific roles of individual lipid species in T2DM are still not well understood [4]. Diets high in calories and low in fiber promote fat accumulation, which contributes to insulin resistance and leads to lipotoxicity in T2DM [4]. These lipid alterations in T2D are largely driven by increased fatty acid flux resulting from insulin resistance [5].

Diabetic dermopathy (DD), also referred to as pigmented pretibial patches or diabetic shin spots, is the most frequently observed skin condition in individuals with diabetes, affecting up to 50% of this population [6]. While its diagnostic significance is debated, some experts regard diabetic dermopathy (DD) as the pathognomonic of diabetes. It is more commonly seen in men and individuals over 50 years of age, with lesion count increasing in relation to age, diabetes duration, and HbA1c levels. Although DD can appear before diabetes is diagnosed, it more often develops later in the disease and is commonly associated with microvascular complications. It frequently precedes neuropathy and retinopathy and is often accompanied by nephropathy [7]. Xerosis, or abnormally dry skin, is another prevalent dermatologic manifestation of diabetes, affecting up to 40% of diabetic individuals [8]. This condition is characterized by scaling, cracking, or a coarse skin texture, most commonly observed on the feet. Obese diabetic patients are reported to experience more pronounced foot hypohidrosis [9].

Cutaneous infections occur in roughly 20% of people with diabetes [10]. In comparison with the general population, diabetic patients are more susceptible to infections and experience them more frequently. Several factors contribute to this increased vulnerability in individuals with poorly controlled diabetes, including vascular and nerve damage, impaired antioxidant defense, leukocyte dysfunction (adherence, chemotaxis, and phagocytosis), and the glucose-rich environment that fosters microbial growth. Additional host risk factors for skin and soft tissue infections in diabetes include persistent hyperglycemia, compromised skin integrity, higher skin pH, neuropathy (sensory or autonomic), mechanical distress or pressure, vascular insufficiency, and immune system impairment [11].

Skin complications are common but often overlooked manifestations in patients with T2DM, and their pattern may vary depending on metabolic conditions such as dyslipidemia. Understanding this relationship can aid in timely diagnosis and comprehensive management of diabetic complications. Moreover, limited data on how dyslipidemia modifies cutaneous signs in T2DM is available in the literature. Therefore, in this study, we compared the dermatological manifestations in patients with T2DM with and without dyslipidemia.

## Materials and methods

This cross-sectional study was conducted at a secondary care hospital, the Sindh Government Hospital, Karachi. A non-probability convenient sampling technique was used. The ethical approval was obtained by the Ethical Review Board of the Sindh Government Hospital with approval number SGH/(2.A/Landhi)1754. The time period of the study was about six months from December 2024 till April 2025. By using open-size OpenEpi 3.01 (Dean AG, Sullivan KM, Soe MM. OpenEpi: Open Source Epidemiologic Statistics for Public Health, Version. www.OpenEpi.com, updated 2013/04/06) software for sample size calculation, the prevalence of skin-related issues in T2DM was 79.2%, as in the previously published study [12]. The calculated sample size was 254 patients. The study involved 280 individuals aged 40-65 years diagnosed with T2DM, distributed into two groups: Group A (with dyslipidemia) and Group B (without dyslipidemia). Participants were excluded if they had type 1 diabetes, hypoglycemia, a history of surgery or chemotherapy, existing dermatological conditions, secondary skin disorders, or significant hepatic or renal dysfunction.

Comprehensive clinical evaluations were carried out to assess glycemic control and diabetes-related complications. Glycemic regulation was primarily assessed using glycosylated hemoglobin (HbA1c) levels, indicating long-term glucose management, alongside postprandial blood glucose levels taken two hours after a meal to evaluate glucose metabolism. Diabetic complications, particularly cardiovascular health, were evaluated through physical assessments, comprising blood pressure and heart rate measurements. Demographic details such as age, sex, height, weight, dietary habits, and smoking status were collected via structured questionnaires (see Appendix 1, 2). The primary investigators involved in data collection were all physicians or medical students. Medical students collected data under the supervision of clinicians and were trained for proper data reproducibility. All patient-identifying information was removed during the data analysis procedure.

The presence of dyslipidemia was determined based on lipid profile results. Diabetes was defined by plasma glucose levels ≥ 7.0 mmol/L, while dyslipidemia was diagnosed if any of the following thresholds were exceeded: total cholesterol (TC) ≥ 5.2 mmol/L, triglycerides (TG) ≥ 1.70 mmol/L, high-density lipoprotein cholesterol (HDL-C) < 1.0 mmol/L, or low-density lipoprotein cholesterol (LDL-C) ≥ 2.59 mmol/L [13]. In addition to demographic, clinical, and biochemical data, all participants underwent detailed dermatological examinations to identify skin conditions commonly linked to T2DM. These evaluations, performed by trained clinicians under standardized settings, focused on identifying xerosis, ichthyosis, diabetic rubeosis, acanthosis nigricans, callosities, bullae, and paresthesias, ensuring accurate and consistent documentation of dermatological findings.

Data was entered and analyzed using IBM SPSS Statistics for Windows, Version 20 (IBM Inc., Armonk, NY, USA). The demographic particulars and dermatological manifestations related to T2DM were documented as frequencies and percentages. Quantitative variables were presented as means and standard deviations. A Chi-square test was used to observe the association of dermatological features in T2DM. Additionally, a Mann-Whitney test was applied to determine the relationship between the means of demographic variables. A p-value < 0.05 was reflected as statistically significant.

## Results

This study included 280 patients with T2DM with and without a history of dyslipidemia. Patients with a history of dyslipidemia (Group A) were significantly older compared to those without dyslipidemia (Group B) (60.27 ± 15.81 vs. 51.13 ± 14.03 years; p<0.001). The mean weight was also higher in Group A (72.11 ± 13.0 kg) than in Group B (66.0 ± 15.8 kg) (p<0.001). Additionally, Group A exhibited a greater mean height (68.09 ± 10.89 inches) compared to Group B (64.56 ± 7.08 inches) (p=0.006). However, a statistically insignificant association was observed in BMI among the two groups (26.21 ± 11.31 vs. 26.75 ± 9.84 kg/m²; p=0.729). Physiological parameters showed notable differences: patients in Group A had significantly higher respiratory rates (20.37 ± 5.92 vs. 16.68 ± 4.60 cycles/min; p<0.001), blood pressure levels (179.57 ± 44.0 vs. 171.03 ± 44.46 mmHg; p=0.007), and heart rates (87.75 ± 10.45 vs. 79.53 ± 11.0 beats/min; p<0.001) compared to those without dyslipidemia. The duration of hypertension was also longer in Group A (5.81 ± 5.99 years) than in Group B (3.7 ± 3.30 years) (p=0.002). In contrast, an insignificant difference was observed in random blood sugar (RBS) levels between the two groups (335.34 ± 96.42 vs. 333.5 ± 113.89 mg/dL; p=0.517), as depicted in Table 1.

**Table 1:** Demographic details, clinical and physiological parameters of patients with T2DM based on history of dyslipidemia (n=280) Data are presented as mean ± standard deviation. The Mann-Whitney test was used for statistical comparisons. p<0.05 is statistically significant.

Variables	Group A, Mean±SD	Group B, Mean±SD	p-value
Age (years)	60.27±15.81	51.13 ±14.03	<0.001
Weight (kg)	72.11±13.0	66.0±15.8	<0.001
Height (Inch)	68.09±10.89	64.56±7.08	0.006
BMI (kg/m^2^)	26.21±11.31	26.75±9.84	0.729
Respiratory rate (cycles/min)	20.37±5.92	16.68±4.60	<0.001
Blood pressure (mmHg)	179.57±44.0	171.03±44.46	0.007
Duration of blood pressure (years)	5.81±5.99	3.7±3.30	0.002
Heart rate (beats/min)	87.75±10.45	79.53±11.0	<0.001
Random blood sugar (RBS) (mg/dL)	335.34±96.42	333.5±113.89	0.517

A highly significant difference was observed in gender distribution; all patients in Group A at 140 (100%) were male individuals, whereas the majority in Group B were female individuals at 124(88.6%) (p<0.001). Socioeconomic status did not differ significantly between the groups (p=0.060), although a higher proportion of Group A patients belonged to the high socioeconomic class 48 (34.3%) versus 31 (22.1%). Regarding comorbidities, no significant association was noted between the groups for the history of hypertension (p=0.695). However, a strong association was observed with smoking status (p<0.001). The types of therapeutic interventions also varied significantly (p=0.001). A larger proportion of Group A patients were using oral hypoglycemic drugs at 65 (46.4%) and insulin at 44 (31.4%) compared to Group B. Interestingly, the combination of diet and insulin was more common in Group B at 26 (18.6%) than in Group A at 8 (5.7%). Duration of diabetes also showed a significant difference between the groups (p<0.001). A larger proportion of Group A had diabetes for more than five years, 47 (33.6%) compared to Group B at 14 (10.0%), whereas a shorter duration (< 1 year) was more common in Group B at 46 (32.9%) compared to Group A at 29(20.7%), as depicted in Table 2.

**Table 2:** Comparison of demographic, lifestyle, and clinical characteristics based on history of dyslipidemia Data are presented as frequency and percentage (n, %). Pearson Chi-square test was used to assess statistical significance. p<0.05 is considered statistically significant.

Variables		Group A, n (%)	Group B, n (%)	Pearson Chi-square	p-value
Gender	Male	140 (100.0%)	16 (11.4%)	222.56	<0.001
	Female	0 (0.0%)	124 (88.6%)		
Socioeconomic status	Low	22 (15.7%)	31 (22.1%)	5.62	0.060
	Middle	70 (50.0%)	78 (55.7%)		
	High	48 (34.3%)	31 (22.1%)		
History of hypertension	Yes	97 (69.3%)	100 (71.4%)	0.15	0.695
	No	43 (30.7%)	40 (28.6%)		
History of smoking	Yes	66 (47.1%)	17 (12.1%)	41.11	<0.001
	No	74 (52.9%)	123 (87.9%)		
Therapy used	Oral hypoglycemic drugs	65 (46.4%)	57 (40.7%)	17.84	0.001
	Insulin	44 (31.4%)	24 (17.1%)		
	Diet only	8 (5.7%)	10 (7.1%)		
	Diet along with oral hypoglycemic drugs	15 (10.7%)	23 (16.4%)		
	Diet along with Insulin	8 (5.7%)	26 (18.6%)		
Duration of diabetes	Less than 1 year	29 (20.7%)	46 (32.9%)	23.48	<0.001
	1 to 5 years	64 (45.7%)	80 (57.1%)		
	More than 5 years	47 (33.6%)	14 (10.0%)		

The distribution of dermatological symptoms among T2DM patients, with and without a history of dyslipidemia revealed that xerosis with fissured skin was significantly more frequent in Group B at 109(77.9%) compared to Group A at 81(57.9%) (p<0.001). Similarly, ichthyosis was more predominant in patients without dyslipidemia at 63 (45.0%) than in those with dyslipidemia at 43 (30.7%), with a statistically significant difference between them (p=0.014). In contrast, an insignificant difference was detected in the occurrence of diabetic rubeosis between the two groups (p=0.324). Acanthosis nigricans was slightly more frequent in Group B at 100 (71.4%) compared to Group A at 86 (61.4%), but the difference was statistically insignificant (p=0.076). Callosities were reported more often in Group B at 48 (34.3%) than in Group A at 31 (22.1%), with the difference reaching statistical significance (p=0.024). The presence of bullae was also significantly greater in Group B at 54 (38.6%) compared to Group A at 25 (17.9%) (p<0.001). Interestingly, paresthesias were reported more frequently in Group A at 86 (61.4%) than in Group B at 69 (49.3%), which was also statistically significant (p =0.041), as depicted in Table 3.

**Table 3:** The dermatological symptoms in T2DM with and without dyslipidemia Data are shown as frequency and percentage (n, %). Pearson Chi-square test was applied to determine associations. p<0.05 is considered statistically significant. T2DM: type 2 diabetes mellitus

Variables		Group A, n (%)	Group B, n (%)	Pearson Chi-square	p-value
Xerosis fissured skin	Yes	81 (57.9%)	109 (77.9%)	12.83	<0.001
	No	59 (42.1%)	31 (22.1%)		
Ichthyosis	Yes	43 (30.7%)	63 (45.0%)	6.07	0.014
	No	97 (69.3%)	77 (55.0%)		
Diabetic rubeosis	Yes	57 (40.7%)	49 (35.0%)	0.97	0.324
	No	83 (59.3%)	91 (65.0%)		
Acanthosis nigricans	Yes	86 (61.4%)	100 (71.4%)	3.14	0.076
	No	54 (38.6%)	40 (28.6%)		
Callosities	Yes	31 (22.1%)	48 (34.3%)	5.09	0.024
	No	109 (77.9%)	92 (65.7%)		
Bulla	Yes	25 (17.9%)	54 (38.6%)	14.83	<0.001
	No	115 (82.1%)	86 (61.4%)		
Paresthesias	Yes	86 (61.4%)	69 (49.3%)	4.17	0.041
	No	54 (38.6%)	71 (50.7%)		

## Discussion

Typically, skin-related manifestations in diabetes develop after the onset of the disease. Research indicates that individuals with T2DM are more prone to developing skin lesions linked to infections, whereas those with type 1 diabetes are more likely to present with autoimmune-related skin conditions [14,15]. Therefore, this study explored the demographic, clinical, and dermatological differences in patients with T2DM with and without a history of dyslipidemia.

The present study showed that individuals with dyslipidemia were significantly older than those without, with mean ages of 60.27 and 51.13 years, respectively. A notable gender difference was also observed, with the dyslipidemic group consisting only of male individuals, while female individuals predominated in the non-dyslipidemic group. Xerosis accompanied by fissured skin was significantly more prevalent in Group B at 109 (77.9%) patients, compared to 81 (57.9%) patients in Group A, with a statistically significant association (p < 0.001). These findings are consistent with earlier research that reported a higher mean age of 63.3 years and a greater proportion of female participants (60 females, 43 males), noting that dermatological symptoms were more prevalent among diabetic individuals over the age of 50. Additionally, previous studies highlighted a high incidence of conditions like diabetic foot (20%), bacterial infections (35%), fungal infections, and cutaneous xerosis (45%) [1]. Similarly, Timshina et al. documented a female-to-male ratio of 1.21:1, reflecting a more balanced gender distribution compared to the marked disparity observed in the present study [16].

Interestingly, another prospective study, which investigated 100 patients comprising 55% male participants and 45% female participants, with a mean age of 52 years, demonstrated that the incidence of cutaneous manifestations increases with patient age, duration of diabetes, and disease severity. Among the dermatological conditions observed, infections were the most frequent, affecting 71% of the participants. Common skin manifestations included skin tags (33%), cherry angiomas (21%), xerosis (19%), acanthosis nigricans (18%), generalized pruritus (12%), xanthelasma palpebrarum (8%), diabetic dermopathy (7%), yellow discoloration of the hands (5%), diabetic thick skin (2%), rubeosis faciei (2%), and granuloma annulare (1%) [17]. These findings were not corroborated with the present study and showed that the patients with a history of dyslipidemia were significantly older compared to those without dyslipidemia (60.27 ± 15.81 vs. 51.13 ± 14.03 years). Dermatological manifestations such as xerosis at 109 (77.9%), acanthosis nigricans at 100 (71.4%), ichthyosis at 63 (45.0%), callosities at 48 (34.3%), and bullae at 54 (38.6%) were more prevalent in higher frequency in diabetic patients without a history of dyslipidemia.

Another study found a significant correlation between dyslipidemia and elevated glycosylated hemoglobin (HbA1c), triglycerides (TG), and low HDL, suggesting poorer metabolic control (p< 0.05) [18]. These findings were corroborated with the present study wherein patients with dyslipidemia showed significantly elevated blood pressure, heart rate, and respiratory rate suggesting higher cardiovascular risk. Additionally, patients in this group had a longer duration of diabetes and hypertension, which is consistent with the natural progression of metabolic complications.

The present study showed that the xerosis was significantly more frequent in the non-dyslipidemic group (109 (77.9%)), which aligns with findings from Galdeano et al. where xerosis was among the most prevalent dermatologic conditions in diabetic patients, with rates of 69% [19]. This consistency indicates that xerosis may be more closely associated with glycemic control, environmental conditions, or the duration of diabetes, rather than with dyslipidemia or lipid profile abnormalities.

Although acanthosis nigricans (AN) did not demonstrate a statistically significant difference between the groups in the present study, it is widely recognized in the literature as an indicator of insulin resistance and increased macrovascular risk. Its prevalence varies among ethnic groups, with reported rates ranging from 1-5% in Caucasians to as high as 13% in Black and Hispanic populations [20,21]. A recent study further identified AN as an independent cardiovascular risk factor, particularly among obese adolescents. This aligns with our finding that AN appeared slightly more often in the non-dyslipidemic group [22].

A study reported by Trihan et al. assessed the incidence of diabetes-associated dermatological manifestations (DADM) in patients with T2DM [23]. Their findings identified diabetic dermopathy (DD) as the most commonly observed skin condition, with a reported occurrence of 17.8% (38 out of 213 patients) [23]. In contrast, the present study found a significantly higher prevalence of DD.

This study was limited by its cross-sectional design, which prevents the establishment of causal relationships between dyslipidemia and dermatological manifestations. Additionally, the findings are based on a single-center population, which may limit generalizability to other settings. Moreover, gender imbalance, sampling bias, and confounding factors, such as the presence of other co-morbidities, like hypertension may affect study findings. 

Further longitudinal, multicenter studies are recommended to explore the causal association between dyslipidemia and specific skin complications in T2DM. Early dermatological screening should be integrated into routine diabetic care to aid in the timely detection and management of skin-related complications.

## Conclusions

This study concluded that the dermatological manifestations were common among T2DM patients, with notable differences based on dyslipidemia status. Patients without dyslipidemia showed a higher prevalence of xerosis, ichthyosis, callosities, and bullae, whereas paresthesias were more frequently observed in those with dyslipidemia. These findings suggest that dyslipidemia may influence the pattern of skin-related complications in diabetic individuals.

## Appendices

Appendix 1

**Table 4:** Questionnaire (section A, demographic and clinical information)

No.	Question	Response
1	Age	________ years
2	Weight	________ kg
3	Height	________ inches
4	Do you have a history of dyslipidemia (high cholesterol/triglycerides)?	☐ Yes ☐ No
5	Do you have a history of hypertension (high blood pressure)?	☐ Yes ☐ No
6	If yes, how long have you had high blood pressure?	________ years
7	Do you have type 2 diabetes mellitus?	☐ Yes ☐ No
8	How long have you had diabetes?	☐ Less than 1 year ☐ 1–5 years ☐ More than 5 years
9	What diabetes treatment are you currently using?	☐ Oral hypoglycemics ☐ Insulin ☐ Diet only ☐ Diet + oral hypoglycemics ☐ Diet + insulin
10	Socioeconomic status	☐ Low ☐ Middle ☐ High
11	Gender	☐ Male ☐ Female
12	Do you smoke or have a history of smoking?	☐ Yes ☐ No
13	Current blood pressure (if known)	________ mmHg
14	Current heart rate (if known)	________ beats/min
15	Respiratory rate (if known)	________ breaths/min
16	Recent random blood sugar (if known)	________ mg/dL

Appendix 2 

**Table 5:** Questionnaire (section B, dermatological manifestations) Leading question: Have you experienced any of the following skin-related symptoms in the past six months?

No.	Symptom	Response
17	Xerosis (dry, cracked, or fissured skin)	☐ Yes ☐ No
18	Ichthyosis (scaly skin)	☐ Yes ☐ No
19	Diabetic rubeosis (reddish skin discoloration, especially on the face)	☐ Yes ☐ No
20	Acanthosis nigricans (dark, velvety patches, usually in folds like neck or armpits)	☐ Yes ☐ No
21	Callosities (thickened skin or calluses on feet)	☐ Yes ☐ No
22	Bullae (fluid-filled blisters)	☐ Yes ☐ No
